# Guideline adherence for cardiometabolic monitoring of patients prescribed antipsychotic medications in primary care: a retrospective observational study

**DOI:** 10.1007/s11096-023-01642-5

**Published:** 2023-09-27

**Authors:** Ruba Azfr Ali, Zahraa Jalal, Jaspal Johal, Vibhu Paudyal

**Affiliations:** 1 Clinical Pharmacy Department, College of Pharmacy, Umm Al-Qura University, Abdya Campus, Prince Sultan Bin AbdulAziz Road, 24381 Makkah, Kingdom of Saudi Arabia; 2 School of Pharmacy, Institute of Clinical Sciences, College of Medical and Dental Sciences, Sir Robert Aitken Institute for Medical Research, University of Birmingham, Birmingham, B15 2TT UK; 3 Dudley Clinical Commissioning Group, Brierley Hill Health and Social Care Centre, Brierley Hill, UK

**Keywords:** Antipsychotics, Cardiometabolic monitoring, Mental disorders, Observational study, Primary care

## Abstract

**Background:**

Despite their known effectiveness, antipsychotics possess significant cardiometabolic adverse event profiles. Guidelines emphasise routine monitoring, however, practices are known to be suboptimal.

**Aim:**

To investigate the level of cardiometabolic monitoring among people prescribed antipsychotic therapy in primary care, and patient-related factors that may influence monitoring patterns.

**Method:**

Data were collected for patients with mental disorders and prescribed antipsychotics at two general practices in England (February 2016–February 2021). The main outcome measures were the proportion of patients with evidence of monitoring for cardiometabolic parameters (body composition, anthropometrics, lipids, glucose outcomes). Regression analysis was used to explore factors predicting monitoring practices.

**Results:**

Data from 497 patients were included. The proportion of patients receiving cardiometabolic monitoring at least once yearly varied across different parameters. Patients were mostly monitored for BP (92.0%), body weight (BMI > 85.0%) and HDL (72.0%), but to a lesser extent for other lipid parameters (non-HDL < 2.0%) and blood glucose (< 2.0%). Ageing (OR:2.0–7.0, *p* < 0.001) and chronic conditions (e.g., CVD and Type 2 DM, *p* < 0.05) were associated with frequent cardiometabolic monitoring. Conversely, antipsychotics with high metabolic risks (olanzapine), patients prescribed antipsychotic polypharmacy (≥ 2 antipsychotics) and cardiometabolic dysregulations (e.g., dyslipidaemias) did not improve monitoring frequencies.

**Conclusion:**

Cardiometabolic health monitoring was generally infrequent, irregular, and did not change in response to abnormal test results or antipsychotic treatment with high cardiometabolic risks, suggesting more efforts need to be made to ensure the guidelines for cardiometabolic monitoring are followed. Future studies should investigate practices by using a large UK primary care database.

**Supplementary Information:**

The online version contains supplementary material available at 10.1007/s11096-023-01642-5.

## Impact statements

- It is important to improve practices in relation to monitoring patients prescribed antipsychotics for cardiometabolic abnormalities.
- Likewise, it is important to promote the role of healthcare professionals who are easily accessible to patients, such as clinical pharmacists. This can help improve the frequency of cardiometabolic monitoring for patients prescribed antipsychotics.

## Introduction

Patients with severe mental disorders, including schizophrenia, affective mood disorder and major depressive disorders, are more prone to premature deaths than the general population [1]. Several aetiologies contribute to the increased mortality in this group, including cardiovascular diseases (CVD) (29.2%) [2]. Antipsychotics are considered the cornerstone in managing a wide range of psychotic disorders [3] along with their use in non-psychotic disorders [4]. Despite their therapeutic effectiveness, antipsychotics, primarily second-generation antipsychotics (SGAs), have a high tendency to cause cardiometabolic abnormalities and hence increase the risk of CVD and other metabolic conditions, particularly metabolic syndrome and type 2 diabetes mellitus (Type 2 DM). Beyond the increased incidence of CVD and metabolic conditions, the stage at which cardiometabolic abnormalities are diagnosed, and the quality of care provided significantly affect the disease course and outcome [5].

In the UK, the National Institute for Health and Care Excellence (NICE) guideline 178 and the Royal College of Psychiatrists (RCP) for managing psychosis and schizophrenia in adults recommended frequent and timely monitoring of cardiometabolic parameters in patients with schizophrenia [6]. Nonetheless, international evidence highlighted persistent deficits in the routine recording of cardiometabolic parameters in patients prescribed antipsychotic therapy. For example, recent evidence assessing CVD screening and treatment in patients with mental disorders reported important disparities in screening practices among patients with mental disorders. This indicates that screening practices for cardiometabolic parameters remain suboptimal, contrary to the guideline recommendations [7, 8].

A review conducted in 2012 on screening practices for metabolic risks in patients prescribed antipsychotics showed an overall monitoring rate of < 50% for all outcomes. The review findings suggest that guideline implementation rarely approaches 100% in actual practice or reaches a level of testing that would be considered ‘appropriate’ for clinical practice [7]. There is a need for updated data describing cardiometabolic monitoring practices in patients prescribed antipsychotic drugs.

### Aim

This study investigated the level of cardiometabolic monitoring in patients prescribed antipsychotic drugs in primary care, and factors that may influence monitoring patterns.

### Ethics approval

This was a secondary data analysis of anonymised patient data that were collected from two primary care general practices in the UK. NHS Research Ethics Committee (REC) approval was obtained through Health Research Authority (HRA), (Ref number: 20/WM/0102), approval date 22 May 2020.

## Method

This was an observational retrospective study examining the frequency of cardiometabolic monitoring in primary care patients prescribed antipsychotics. The study was reported as per the STrengthening the Reporting of Observational studies in Epidemiology (STROBE) statement for observational studies [9].

### Eligibility criteria

This study included patients prescribed chronic antipsychotic therapy for mental disorders, primarily schizophrenia-related disorders, affective mood disorders, and depression. Chronic antipsychotic therapy was defined as a prescription for antipsychotics for at least 12 months. As the study focused on patients on chronic antipsychotic therapy, records for patients who had only one record of antipsychotic prescription (e.g., prn indications) were excluded regardless of the date of prescription issue. Also, patients who stopped antipsychotic treatment or left the site were excluded.

A sample size of 150 was calculated based on the reported prevalence of monitoring using the formula for cross-sectional studies [10]:

$$N = 4pq/d^{2}$$

where N: the calculated sample size. p: expected proportion in the population reported in the literature. q: i. When p is in percentage terms: (100-p), ii. When p is in decimal terms: (1-p). d: The precision of the estimate. This could either be the relative precision or the absolute precision.

Since the study had multiple outcomes, the size of the lowest reported outcome with the largest reported prevalence was selected. As an example, plasma lipids (27.0% to 69.7%) had a minimum reported prevalence range, and the minimum limit (27.0%) was used. With a relative precision of 27.0% (calculated precision = 7.8), the study would be able to detect the true monitoring level if the actual prevalence was 23.0% or higher (half the value of relative precision on either side of the selected ‘*p*’ (± 3.8%): 23% to 30.8%).

### Measurements of outcome variables

The primary outcome was the proportion (%) of patients who presented with records of cardiometabolic parameters at any time during the study period. The outcome parameters were cardiometabolic health. These parameters included (1) Body weight: body mass index (BMI) and waist circumference (WC); (2) Blood pressure (BP); (3) Blood glucose (BG): fasting blood glucose (FBG) and glycosylated haemoglobin (HbA1c); (4) Lipid profile: high-density lipoprotein (HDL) and (non-HDL).

The proportion was estimated based on the recorded information in the database. For cardiometabolic monitoring, positive observations were interpreted based on the actual data recording: the date of order entry, medical code, and corresponding laboratory findings. To assess monitoring density, monitoring parameters were divided into three levels: less frequent, standard, and more frequent. This classification was based on the NICE guidance for physical health monitoring in schizophrenia, which recommends at least one monitoring instance per year for patients on chronic antipsychotic treatment [6]. The mean frequency of monitoring for each outcome variable was calculated over three years. The density of cardiometabolic monitoring was defined by applying the following scenarios and linked descriptions: (1)‘Less frequent’ when there was a low number of tests/year [mean frequency < 1]; (2)‘Standard monitoring’ was defined by the mean frequency = 1; (3)‘More frequent monitoring’[mean frequency > 1] indicated a high number of tests conducted over 3 years. A cut-off point equal to (1) was used as a factor to represent standard monitoring where the patients received only one monitoring per year.

### Covariates

Factor variables corresponded to participants’ demographics and clinical variables that independently affected the study outcomes. These factors were selected according to the NICE guideline definition of cardiovascular risk factors (i.e., BMI, BP, smoking status, BG, lipid profiles, and family history of CVD) [11]. To assess the predictive effect of antipsychotics in cardiometabolic monitoring, factors related to antipsychotic treatment were used as covariates in the regression model. These factors included the type of antipsychotic drug, antipsychotic polypharmacy (monotherapy and polypharmacy) and known cardiometabolic profile for individual antipsychotic drugs.

### The data source

This observational study analysed data that were collected from a local Clinical Commissioning Group (CCG) of the National Health Service (NHS). A website, Egton Medical Information Systems (EMIS), is used by the practice to collect clinical routine data. The EMIS web consists of codes from the Dictionary of Medicines and Devices coding system (dm + d codes) that is based on the British National Formulary (BNF) coding system. The EMIS also uses READ clinical codes, which include medical and non-medical terms categories.

### Data extraction procedure

A standardised extraction tool was developed to collect data from the EMIS web. The tool mimicked the information layout in the EMIS system and number-based variables (e.g., 0 = negative, 1 = positive) were used to facilitate extraction by ensuring an easy-to-read layout and minimising text amount. Eligible patients’ records were identified by matching the clinical READ code (E10) for schizophrenia and psychosis with the dm + d codes for antipsychotic drugs (Supplemental material S1). Patients were considered on chronic antipsychotic treatment if they had two consecutive treatment periods. The first time point represented recent prescriptions (defined as records for patients receiving antipsychotics within 12 months from the extraction date). The second point fulfilled treatment chronicity, defined as repeated records of antipsychotic prescriptions that extend at least 36 months before extraction to ensure that all included patients had matched prescription and monitoring records.

In the presence of multiple antipsychotic prescriptions, the history of previous prescriptions for the two antipsychotics was used to make the distinction between main and add-on antipsychotics. For the cardiometabolic monitoring pattern, history records for matched cardiometabolic outcomes (records for the past 3 years) were checked. The 3-year window (February 2018–February 2021) was applied to capture long-interval monitoring patterns for some parameters. The extraction was conducted by a delegated, experienced data extractor in the NHS Trust who had access to the data using an extraction tool that was designed and piloted before the final extraction. The data were extracted from the database into spreadsheets and handed over to the study team.

### Statistical analysis plans

Proportion values were computed for categorical variables via descriptive statistics. Chi-square (χ^2^) and Fisher’s exact tests were conducted to compare the differences among frequency counts for different cardiometabolic outcomes. Firth logistic regression was performed to predict the influence of clinical factors on the likelihood of having cardiometabolic monitoring at least once during the study period [12]. All statistical analyses were performed using Stata software 17.0.

## Results

**Fig. 1:**
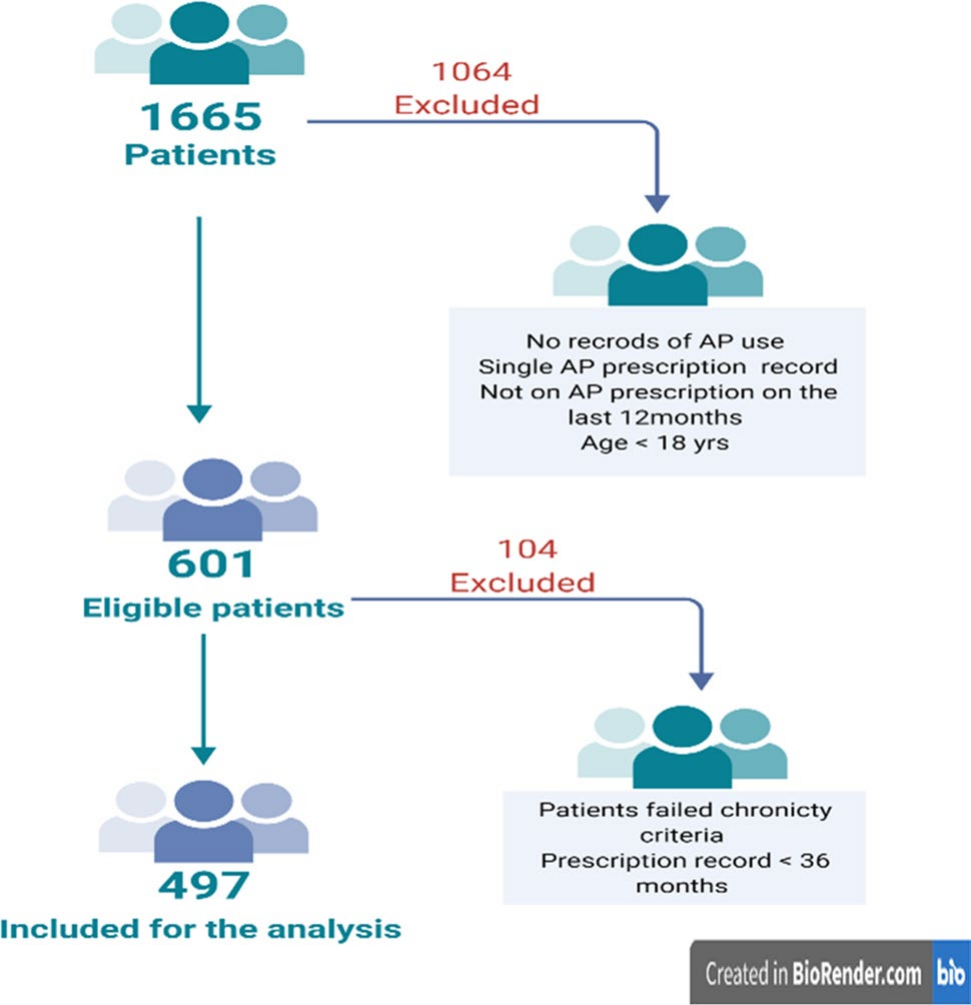
Selection process for patients’ records. APs: antipsychotics. Created by BioRender.com [13]

The number of patients prescribed antipsychotic treatment for at least 12 months was 1665. After applying the inclusion criteria, 497 records were included for the final analysis. Figure 1 shows the records selection process [13].

### Demographic and clinical information

A total of 51.3% (n = 255) of patients were male, and 48.7% (n = 242) were female. Almost one quarter, 21.0% (n = 103), were aged between 41 and 50 years old. The mean age was 41.9 years (SD = 14.3 years, range 18–95 years). Around half (n = 266, 53.5%) had a family history of CVD and 39.6% (n = 197) were smokers. The demographic and clinical characteristics are presented in Table 1.

**Table 1:** Demographic and clinical characteristics (Total = 497)

Characteristics	n (%)
Gender	
Female	242 (48.6)
Male	255 (51.3)
Total	497
Age (years)	
17–30	71 (14.2)
31–40	88 (17.7)
41–50	103 (20.7)
51–60	97 (19.5)
61–70	63 (12.6)
71–80	49 (9.8)
≥ 81	26 (5.2)
Total	497
Smoking status	
Non-smoker	194 (39.6)
Ex-smoker	98 (20.0)
Current smoker	197 (40.2)
Total	489 (98.3)
Missing smoking records	8 (1.6)
Family history of CVD	
No	231 (46.4)
Yes	266 (53.5)
Total	497
Known diagnosis of CVD	
No	345 (69.4)
Yes	152 (30.5)
Total	497
Known diagnosis of DM	
No	454 (91.3)
Yes	43 (8.6)
Total	497
Current anti-diabetic medicines	
No	451 (90.7)
Yes	46 (9.2)
Total	497
Current anti-hypertensive medicines	
No	434 (87.3)
Yes	63 (12.6)
Total	497
Current lipid-lowering agents	
No	360 (72.4)
Yes	137 (27.5)
Total	497
Blood pressure (mmHg)	
Low BP (Systolic:60–90 or Diastolic: < 60)	14 (2.8)
Normal BP (Systolic: 90–120 or Diastolic: 60–80)	133 (26.7)
Pre-HTN (Systolic: 120–140 or Diastolic: 80–90)	238 (47.8)
HTN (Systolic > 140 or Diastolic > 100)	68 (13.6)
Total	453 (91.1)
†Observations not recorded	44 (8.8)
BMI (kg/m^2^)	
Underweight (< 18.5)	8 (1.6)
Normal (18.5–24.9)	103 (20.7)
Overweight (25.0–29.9)	124 (24.9)
Obese 1 (30.0–34.9)	113 (22.7)
Obese 2 (35.0–39.9 and above)	83 (16.7)
Total	431 (86.7)
†Observations not recorded	66 (13.2)
Lipid profile	
HDL (mmol/L)	
Normal (≥ 1 mmol/L)	185 (37.2)
Dyslipidaemia (< 1 mmol/L)	162 (32.6)
Total	347 (69.8)
†Observations not recorded	150 (30.1)
Non-HDL (mmol/L)	
Normal (≤ 4 mmol/L)	99 (19.9)
Dyslipidaemia (> 4 mmol/L)	227 (45.6)
Total	326 (65.6)
†Observations not recorded	171 (34.4)

†Corresponds to non-recorded patients

*BMI* body mass index, *WC* waist circumference, *BP* blood pressure, *HbA1c* glycosylated haemoglobin, *HDL* high-density lipoprotein, *CVD* cardiovascular, *DM* diabetes mellitus, *HTN* hypertension

Regarding cardiometabolic health, 30.5% (n = 152) had a diagnosis of CVD. Over 70.0% (n = 382) had at least one abnormal cardiometabolic outcome. Nearly more than half (n = 320, 64.3%) were classified as obese according to their BMI score. Around half were considered pre-hypertensive (n = 238, 47.8%) and a small proportion was diagnosed with high BP (n = 68, 15%), and of this proportion, 12.6% (n = 63) had records for anti-hypertensive medications (Table 1). The nature of antipsychotic therapy and cardiometabolic risks associated with individual antipsychotics are described in Table 2.

**Table 2:** Characteristics of prescribed antipsychotic therapy (N = 497)

Antipsychotic drug class	n (%)
**First-generation antipsychotics (FGAs)**	**98 (19.7)**
Amisulpride	15 (3.02)
Chlorpromazine	9 (1.81)
Flupentixol	3 (0.6)
Haloperidol	8 (1.61)
Pimozide	1 (0.2)
Promazine	52 (10.5)
Sulpiride	5 (1.0)
Trifluoperazine	1 (0.2)
Zuclopenthixol	4 (0.8)
**Second-generation antipsychotics (SGAs)**	**399 (80.2)**
Aripiprazole	75 (15.1)
Clozapine	8 (1.6)
Olanzapine	68 (13.6)
Quetiapine	155 (31.1)
Risperidone	93 (18.7)
Psychotropic polypharmacy*	
Yes	400 (80.5)
Number of other prescribed psychotropic agents	
1	175 (43.8)
2	127 (32)
3–6	98 (25)
Categories of psychotropic medications prescribed	
Anti-anxiety medications	30 (7.5)
Antipsychotic medications	28 (7.0)
Antidepressant medications	331 (82.7)
Mood stabilizers	109 (27.2)
CNS stimulants	14 (3.5)
Other psychiatry Agents (Anti-abuse medications)	4 (1.0)
Associated cardiometabolic risks with APs use (497)	
Drug risk/extent of weight gain	
Limited/No data	61 (12.2)
Neutral/low	80 (16.1)
Moderate	256 (51.5)
High	100 (20.1)
Drug risk/extent of hyperglycaemia/diabetes	
Limited/No data	10 (2.10)
Minimal/low	154 (30.9)
Moderate	248 (49.9)
High	85 (17.1)
Drug risk/extent of dyslipidaemias	
Limited/No data	1(0.2)
Neutral/Low	172 (34.6)
Moderate/high	324 (65.1)

The bold font represnts the main antipsychotic drug classes

*≥ 2 psychotropic drugs

Approximately 80.5% (n = 400) were prescribed concomitant psychotropic medications, including antipsychotic polypharmacy (n = 28, 7.0%) (Table 2). Antidepressant medications were the most concomitant psychotropic medications (n = 331, 82.8%). A large proportion of patients (n = 366, 74.0%) were prescribed antipsychotic medications with known cardiometabolic risks. Concerning antipsychotic therapy, most patients were prescribed second-generation antipsychotics (n = 399, 80.3%), with quetiapine (n = 155, 31.1%) and risperidone as the most prescribed antipsychotic medications (n = 93, 18.7%). Of the 366 patients using highly cardiometabolic labile antipsychotics, commonly prescribed were quetiapine (n = 155, 31.1%), risperidone (n = 93, 18.7%), and olanzapine (n = 68, 13.6%). Among patients prescribed antipsychotic agents with neutral cardiometabolic effects, aripiprazole was the most commonly prescribed drug (n = 75, 15.1%).

### Cardiometabolic monitoring profile

**Fig. 2:**
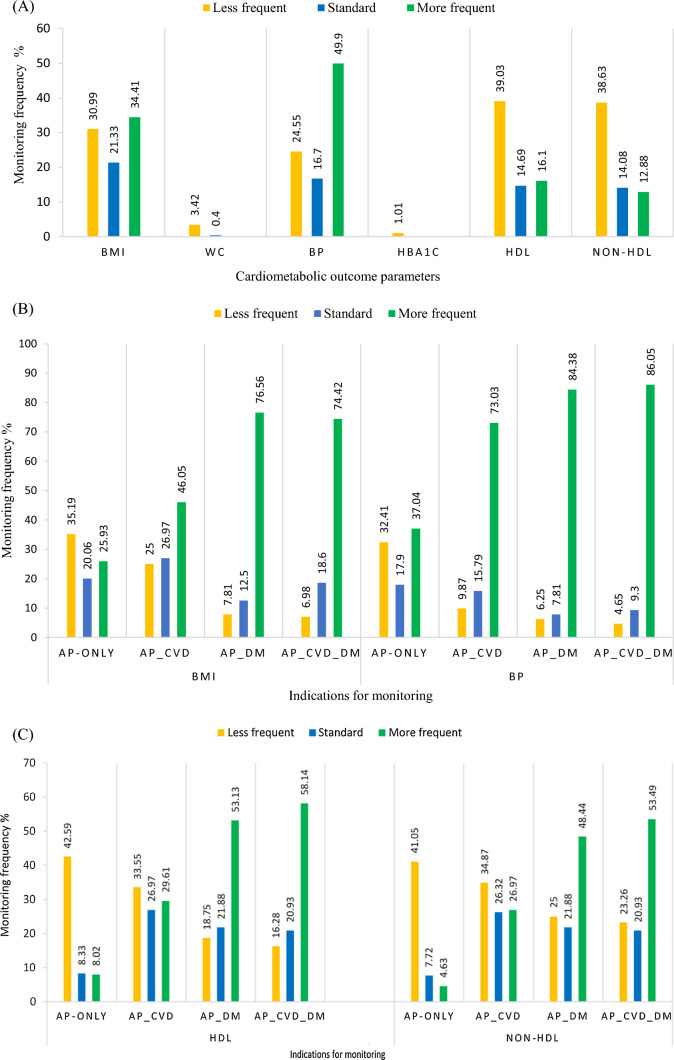
Overall monitoring density for cardiometabolic testing of patients receiving antipsychotic medication in the last 3-years (**A**) Cardiometabolic monitoring density for body compositions and blood pressure (**B**), and lipid profiles (**C**) by different indications. Abbreviations: *BMI* body mass index, *WC* waist circumference, *BP* blood pressure, *HBA1C* glycosylated haemoglobin, *HDL* high-density lipoprotein, *non-HDL* non high-density lipoprotein, *AP* antipsychotic, *CVD* cardiovascular diseases, *DM* diabetes mellitus. Data represent the proportion (%) of monitored participants. Exposure characteristics: AP-only: Antipsychotic drug only (no other comorbidities), Antipsychotic with pre-existing comorbidities (CVD or DM or both)

Initially, data were classified by monitoring status. Accordingly, patients were stratified into monitored and non-monitored subpopulations based on the presence of ≤ 1 monitoring event (last 3 years). To assess the frequency of monitoring for different indications, patients were sub-classified further according to the presence of extra-indications for cardiometabolic monitoring into (i) AP-only group: Antipsychotic drug only (no other comorbidities); (ii) AP-CVD group: Antipsychotic with pre-existing comorbidities (CVD); (iii) AP-DM group: Antipsychotic with pre-existing comorbidities (DM); (iv) AP-CVD-DM group: Antipsychotic with pre-existing comorbidities (both CVD and DM). The frequency of monitoring for various indications is described in Fig. 2 (A, B and C).

None of the patients were fully monitored for all parameters according to NICE/RCP guidelines. BP and BMI were documented in more than 86.6% (n = 430), while more than 96.0% (n = 477) did not have WC measurements. Similarly, HbA1c was recorded in only 1.0% of the patients, yet none had FBG records. As for the monitoring density, standard monitoring was achieved in only approximately 20.0% (n = 103). Comorbidities were positively associated with frequent monitoring for different cardiometabolic outcomes compared to the AP-only group (*p* < 0.001) (see Fig. 2).

### Monitoring density for cardiometabolic parameters by associated cardiometabolic risks of antipsychotics

Of the entire sample, 415 were prescribed antipsychotics with moderate/high cardiometabolic risks (Supplemental material S2). Although patients who were prescribed antipsychotics with moderate/high risks of weight gain (n = 356, 71.6%) received frequent body weight monitoring, the differences were non-significant (n = 110), χ^2^ (df = 9) = 12.5, *p* = 0.2. Likewise, the proportions of patients who received standard/more frequent lipid monitoring (26.0%) in patients receiving antipsychotics with a known risk of dyslipidaemia significantly differed from ones who were infrequently monitored (39.0%), χ^2^ (df = 6) = 25.2, *p* < 0.001.

### Predictors of cardiometabolic monitoring in people prescribed antipsychotics

The analysis showed that age > 30 years (OR:2.0–7.0, *p* < 0.001) and a family history of CVD (OR > 1.8, *p* < 0.001) significantly affected cardiometabolic monitoring. There was also strong evidence that the odds of receiving lipid monitoring increased with a smoking history and increased diabetes risks. Similarly, patients receiving medications for comorbidities (lipid-lowering and antidiabetic drugs) were more likely to receive plasma lipid monitoring. Conversely, prescribing antipsychotics with documented cardiometabolic risks (quetiapine) was not predictive of cardiometabolic monitoring. Similarly, patients on psychotropic polypharmacy were monitored more often for body weight and BP, but the increase was non-significant (*p* = 0.8) (Table 3).

**Table 3:** Univariate analysis of body weight (BMI), blood pressure and lipids monitoring trends against different regressors

Outcome	†Model 1 BMI		Model 2 Blood pressure		Model 3 HDL		Model 4 Non-HDL	
Factor variables	OR	95% CI	OR	95% CI	OR	95% CI	OR	95% CI
Gender								
Female (reference)	–	–	–	–	–	–	–	–
Male	0.6	0.4–1.1	0.64	0.4–1.1	0.8	0.52–1.1	0.74	0.51–1.1
Age (Years)								
17–30 (reference)	–	–	–	–	–	–	–	–
31–40	2.33	1.1–1.6	1.28	0.54–3	2.47	1.3–4.7	2.34	1.2–4.5
41–50 51–60 61–70 71–80 ≥ 81	4.5 5.45 3.42 3 7.7	1.9–10.4 2.2–13.4 1.3–8.5 1.2–7.9 1.4–43.1	2.7 2.9 1.9 6.8 11.1	1.03–7.1 1.1–8.1 0.7–5.1 1.2–38.5 0.6–195.1	5.46 8.3 13.5 16.7 20.2	2.8–10.5 4.1–16.7 5.6–32.2 6.1–46 5–81.3	5.2 6.3 10.7 15.7 23	2.7–9.9 3.2–12.4 4.8–24.2 6–41.3 5.7–92.9
Family history of CVD								
No (reference)	–	–	–	–	–	–	–	–
Yes	2.7	1.6–4.8	3	1.5–5.7	2	1.4–3.1	1.87	1.3–2.7
Smoking status								
Non-smoker (reference)	–	–	–	–	–	–	–	–
Ex-smoker Current smoker	1.14 0.9	0.54–2.4 0.5–1.5	1.3 1.1	0.56–3.2 0.5–2.1	1.6 1.04	0.8–2.6 0.7–1.6	1.86 0.95	1.1–3.2 0.63–1.4
CV Risk by QRISK score								
High (Reference) Low	– 1.04	– 0.5–2.3	– 0.82	– 0.34–1.9	– 0.31	– 0.17–0.57	– 0.33	– 0.2–0.57
Diabetes risk								
No (reference) Yes	– 1.87	– 0.6–5.7	– 1.16	– 0.4–3.6	– 3.2	– 1.3–8.21	– 4.03	– 1.6–10.1
Current anti-hypertensive medicines								
No (reference) Yes	– 7.38	– 1.4–38.1	– 14.4	– 0.8–237.9	– 4.4	– 1.9–10.1	– 4.6	– 2.1–10.1
Current antidiabetic medicines								
No (reference) Yes	– 2.96	– 0.81–10.9	– 3.23	– 0.6–16.9	– 3.6	– 1.4–8.9	– 5.56	– 2.1–14.9
Current lipid-lowering medicines								
No (reference) Yes	– 4.07	– 1.8–9.4	– 3.7	– 1.4–10.1	– 13.5	– 5.9–30.5	– 12.6	– 6.1–26.02
Psychotropic polypharmacy*								
No (reference) Yes	– 1.03	– 0.5–1.9	– 1.27	– 0.6–2.6	– 0.88	– 0.5–1.42	– 0.8	– 0.5–1.3
Weight gains associated risks with ATPs								
Limited/No data (reference) Neutral/Low Moderate High	– 3.19 1.7 1.9	– 1.2–8.6 0.9–3.5 0.84–4.5	– 3.51 1.4 2	– 0.9–12.8 0.6–3.1 0.7–5.6	– 1.5 0.6 1	– 0.7–3.4 0.3–1.2 0.5–2.1	– 2.1 1 1.5	– 0.98–4.3 0.5–1.5 0.8–2.9
Hyperglycaemia associated risks with ATPs								
Limited/No data (reference) Minimal/ Low Moderate High	– 2.04 1.8 1.9	– 0.5–9 0.4–7.8 0.41–8.8	– 3.4 2.5 4.3	– 0.7–15.3 0.6–11.1 0.8–22.4	– 1 0.5 0.73	– 0.24–4.5 0.12–2.1 0.2–3.3	– 1.3 0.65 1.1	– 0.4–4.9 0.2–2.3 0.3–4.2
Dyslipidaemia associated risks with ATPs								
Limited/No data (reference) Neutral/Low Moderate/High	– 2.22 2.1	– 0.08–56.4 0.13–83.8	– 3.38 3.33	– 0.13–86.7 0.13–83.8	– 1.16 0.62	– 0.1–29.1 0.02–15.5	– 0.91 0.52	– 0.04–22.6 0.02–13.1
Individual ATPs with known high risks								
Olanzapine	0.9	0.4–2.2	2.1	0.6–7.5	0.73	0.4–1.4	0.9	0.5–1.6
Quetiapine	0.8	0.43–1.5	1.1	0.49–2.3	0.47	0.29–0.8	0.46	0.3–0.7
Risperidone	1.2	0.55–2.7	0.65	0.3–1.4	0.52	0.3–0.9	0.59	0.4–1.02

^†^It was not feasible to model some outcome variables (e.g., WC and HbA1c) due to the limited number of positive observations. ** ≥ 2 psychotropic drugs. *BMI* Body mass Index, *HDL* High-density lipoprotein, *Non-HDL* non High-density lipoprotein, *95% CI* Confidence interval, *OR* Odds Ratio, *CVD* Cardiovascular disease, *CV* Cardiovascular, *ATPs* Antipsychotics

## Discussion

### Key findings

The findings showed considerable variation in cardiometabolic monitoring frequencies in patients with mental disorders prescribed antipsychotics. Regarding the monitoring frequency level, out of the seven parameters that were covered in this study (BMI, WC, BP, FBG, HbA1c, HDL and non-HDL), BP and BMI were mostly recorded. However, standard monitoring was only achieved at 20.0% for BMI, BP, and plasma lipids indicating that routine monitoring of antipsychotics for metabolic parameters in primary care was not conducted according to NICE guidelines [6].

Previous investigations found similar results. In a UK study, the authors found that physical assessments for BMI and BP were mostly recorded. The study also found that biochemical measures were obtained for half of the patients (BG was recorded for < 60.0% and lipid profiles were recorded for 3.5–23.0%) [14]. Similarly, a baseline audit aiming to assess the frequency of metabolic monitoring in outpatients with schizophrenia receiving antipsychotics in Malaysia revealed an extremely low initial level of metabolic monitoring (< 20.0%), which was significantly raised following intervention [15]. Another study assessing metabolic monitoring provided further evidence of inconsistent frequency of metabolic rate monitoring in psychiatric patients in South India. Initially, 99.7% had their BP, while 47.0% had their FBS levels recorded. Over time, monitoring rates decreased for BP but remained the same for FBS [16].

The regression model suggests that among the ten patient factors assessed for association with cardiometabolic monitoring, seven variables best predicted monitoring frequency. These factors included age, pre-existing metabolic conditions, CVD family history, risk of CVD and diabetes, and corresponding treatments. Frequent cardiometabolic monitoring related to age has been generally observed in related investigations [17, 18]. Yet, evidence addressing factors affecting cardiometabolic monitoring performance in primary mental health services is limited.

While our study sample consisted of few prediabetic patients, the number of BG records was generally low. Even among obese patients (BMI ≥ 25 kg/m^2^) without diabetes, BG monitoring was not adequately performed. Our findings contrast with previous evidence highlighting a potential association between conditions including diabetes and dyslipidaemia with cardiometabolic monitoring patterns in patients with mental disorders, suggesting that such comorbidities make patients more prone to monitoring. For example, data from 2010 viewed factors including pre-existing dyslipidaemia and diabetes as significant predictors for recording all metabolic syndrome diagnostic components among antipsychotic users [17].

### Interpretation

Our findings showed that central obesity measurements were barely conducted, which is consistent with the literature. The results of a study analysing data from a metropolitan mental health hospital reported an extremely low level of WC monitoring, being recorded in only 7.0% of screened records [19]. Similarly, previous work showed that WC was not recorded in the reviewed files, despite interventions applied for this purpose [14, 20]. Weight gain, especially when manifested as central obesity, remains a significant long-term health issue in patients taking antipsychotics, even with normal BMI scores [21].

This study revealed a low rate of BG monitoring of less than 2.0%, despite diabetes being independent of weight gain. Previous work reported various levels of BG monitoring from 15.0 to 46.0% [22, 23], which is markedly higher than our recorded rate. The observed increase in monitoring patterns reported in previous studies may have resulted from either the close monitoring provided by specialised centres or interventions (a predesigned monitoring form). However, the reasons for this low monitoring rate of the BG observed in this study are not fully clear. Furthermore, in this study, most patients were prescribed second-generation antipsychotics, which appeared to have no impact on metabolic monitoring. Our findings showed that patients were more likely to receive cardiometabolic monitoring for co-existing morbidities, which could be considered more important determinants of cardiometabolic monitoring among patients prescribed antipsychotics.

We found no correlation between the use of antipsychotics with known metabolic liabilities (quetiapine, risperidone, or olanzapine) and cardiometabolic monitoring, which is inconsistent with the recommendations of NICE/RCP emphasising preferential monitoring based on the administered antipsychotic. Similar reports showed that the use of second-generation antipsychotics was significantly associated with low HbA1c testing, calling for increased metabolic screening among such patients [23–25].

### Strengths and weaknesses

The use of a pre-piloted data extraction tool reduced data sampling bias. Also, the sample size was adequate to ensure the precision of the observed results and estimations. However, this study has several limitations related to the used design. The study design was retrospective hence that relied on the accuracy of data recording and data coding in EMIS web. The generalisability of the results may be limited due to the small number of participating sites. 

### Future research

Future research should target health promotion strategies in primary care for monitoring and follow-up interventions, mainly for unrecorded variables, e.g., WC and BG. Routine physical health monitoring, referrals, and treatment for patients with schizophrenia, the current system of fragmented mental and physical health services could be transformed into a system focusing on early interventions. The under-monitoring observed in this study can be explained by barriers impacting efficient monitoring for cardiometabolic abnormalities. Addressing these barriers would improve cardiometabolic monitoring practices for antipsychotics in primary care. Also, qualitative research targeting prospective healthcare providers in primary care regarding the importance of cardiometabolic monitoring among antipsychotic users is warranted.

## Conclusion

This study provides evidence of suboptimal routine monitoring of cardiometabolic consequences in patients prescribed antipsychotics. Furthermore, it quantifies the extent of the under-recognition of modifiable risk factors for CVD such as central obesity, hypertension, dyslipidaemia, and hyperglycaemia among patients on antipsychotic therapy. It emphasises the lack of translation of guidelines into action for the prevention, early detection, and treatment of these risk factors in a particularly high-risk group of individuals. There is a need to investigate individual and organisational factors influencing the adoption of monitoring guidelines in primary care for patients prescribed antipsychotic medications.

### Supplementary Information

Below is the link to the electronic supplementary material.

Supplementary file1 (PDF 269 KB)
