# Supplementary material for: Guideline adherence for cardiometabolic monitoring of patients prescribed antipsychotic medications in primary care: a retrospective observational study
Source: Int J Clin Pharm. 2023 Sep 27;45(5):1241–51. doi: 10.1007/s11096-023-01642-5 (PMC10600311; doi:10.1007/s11096-023-01642-5)
Supplement: Supplementary file 1 — Supplementary file1 (PDF 269 KB) [file 11096_2023_1642_MOESM1_ESM.pdf]

Supplemental material S1

Table S1-1: Clinical READ codes list for mental conditions used for the data extraction

Medical code	Description
E102.00	Catatonic schizophrenia
E102000	Unspecified catatonic schizophrenia
E102100	Subchronic catatonic schizophrenia
E102200	Chronic catatonic schizophrenia
E102300	Acute exacerbation of subchronic catatonic schizophrenia
E102400	Acute exacerbation of chronic catatonic schizophrenia
E102500	Catatonic schizophrenia in remission
E102z00	Catatonic schizophrenia NOS
E10..00	Schizophrenic disorders
E100.00	Simple schizophrenia
E100.11	Schizophrenia simplex
E100000	Unspecified schizophrenia
E100100	Subchronic schizophrenia
E100200	Chronic schizophrenic
E100300	Acute exacerbation of subchronic schizophrenia
E100400	Acute exacerbation of chronic schizophrenia
E100500	Schizophrenia in remission
E100z00	Simple schizophrenia NOS
E101.00	Hebephrenic schizophrenia
E101000	Unspecified hebephrenic schizophrenia
E101100	Subchronic hebephrenic schizophrenia
E101200	Chronic hebephrenic schizophrenia
E101300	Acute exacerbation of subchronic hebephrenic schizophrenia
E101400	Acute exacerbation of chronic hebephrenic schizophrenia
E101500	Hebephrenic schizophrenia in remission
E101z00	Hebephrenic schizophrenia NOS
E103.00	Paranoid schizophrenia
E103000	Unspecified paranoid schizophrenia
E103100	Subchronic paranoid schizophrenia
E103200	Chronic paranoid schizophrenia

Medical code	Description
E103300	Acute exacerbation of subchronic paranoid schizophrenia
E103400	Acute exacerbation of chronic paranoid schizophrenia
E103500	Paranoid schizophrenia in remission
E103z00	Paranoid schizophrenia NOS
E104.00	Acute schizophrenic episode
E104.11	Oneirophrenia
E105.00	Latent schizophrenia
E105000	Unspecified latent schizophrenia
E105100	Subchronic latent schizophrenia
E105200	Chronic latent schizophrenia
E105300	Acute exacerbation of subchronic latent schizophrenia
E105400	Acute exacerbation of chronic latent schizophrenia
E105500	Latent schizophrenia in remission
E105z00	Latent schizophrenia NOS
E106.00	Residual schizophrenia
E106.11	Restzustand - schizophrenia
E107.00	Schizo-affective schizophrenia
E107.11	Cyclic schizophrenia
E107000	Unspecified schizo-affective schizophrenia
E107100	Subchronic schizo-affective schizophrenia
E107200	Chronic schizo-affective schizophrenia
E107300	Acute exacerbation subchronic schizo-affective schizophrenia
E107400	Acute exacerbation of chronic schizo-affective schizophrenia
E107500	Schizo-affective schizophrenia in remission
E107z00	Schizo-affective schizophrenia NOS
E10y.00	Other schizophrenia
E10y.11	Cenesthopathic schizophrenia
E10y000	Atypical schizophrenia
E10y100	Coenesthopathic schizophrenia
E10yz00	Other schizophrenia NOS
E10z.00	Schizophrenia NOS
146H.00	H/O: psychosis

Medical code	Description
212T.00	Psychosis, schizophrenia + bipolar affective disord resolved
212X.00	Psychosis resolved
2229.13	O/E - senility - no psychosis
8G13100	CBTp - cognitive behavioural therapy for psychosis
8HHs.00	Referral to psychosis early intervention service
E00y.11	Presbyophrenic psychosis
E011000	Korsakov's alcoholic psychosis
E011100	Korsakov's alcoholic psychosis with peripheral neuritis
E01y.00	Other alcoholic psychosis
E01yz00	Other alcoholic psychosis NOS
E01z.00	Alcoholic psychosis NOS
E02z.00	Drug psychosis NOS
E03y300	Unspecified puerperal psychosis
E040.11	Korsakoff's non-alcoholic psychosis
E04z.00	Chronic organic psychosis NOS
E110300	Single manic episode, severe without mention of psychosis
E110400	Single manic episode, severe, with psychosis
E111300	Recurrent manic episodes, severe without mention psychosis
E111400	Recurrent manic episodes, severe, with psychosis
E112300	Single major depressive episode, severe, without psychosis
E112400	Single major depressive episode, severe, with psychosis
E113300	Recurrent major depressive episodes, severe, no psychosis
E113400	Recurrent major depressive episodes, severe, with psychosis
E114300	Bipolar affect disord, currently manic, severe, no psychosis
E114400	Bipolar affect disord, currently manic,severe with psychosis
E115300	Bipolar affect disord, now depressed, severe, no psychosis
E115400	Bipolar affect disord, now depressed, severe with psychosis

Medical code	Description
E116300	Mixed bipolar affective disorder, severe, without psychosis
E116400	Mixed bipolar affective disorder, severe, with psychosis
E117300	Unspecified bipolar affective disorder, severe, no psychosis
E117400	Unspecified bipolar affective disorder,severe with psychosis
E11zz00	Other affective psychosis NOS
E121.00	Chronic paranoid psychosis
E12z.00	Paranoid psychosis NOS
E130.00	Reactive depressive psychosis
E131.00	Acute hysterical psychosis
E134.00	Psychogenic paranoid psychosis
E13y100	Brief reactive psychosis
E13z.00	Nonorganic psychosis NOS
E141.00	Disintegrative psychosis
E141z00	Disintegrative psychosis NOS
E14y100	Borderline psychosis of childhood
E14z.00	Child psychosis NOS
E1z..00	Non-organic psychosis NOS
Eu02z12	[X] Presenile psychosis NOS
Eu02z15	[X] Senile psychosis NOS
Eu03.11	[X]Korsakov's psychosis, nonalcoholic
Eu04.13	[X]Acute / subacute infective psychosis
Eu05212	[X]Schizophrenia-like psychosis in epilepsy
Eu05y11	[X]Epileptic psychosis NOS
Eu0z.11	[X]Organic psychosis NOS
Eu0z.12	[X]Symptomatic psychosis NOS
Eu10514	[X]Alcoholic psychosis NOS
Eu10611	[X]Korsakov's psychosis, alcohol induced
Eu22011	[X]Paranoid psychosis
Eu23012	[X]Cycloid psychosis
Eu23112	[X]Cycloid psychosis with symptoms of schizophrenia
Eu23312	[X]Psychogenic paranoid psychosis
Eu23z11	[X]Brief reactive psychosis NOS

Medical code	Description
Eu23z12	[X]Reactive psychosis
Eu25011	[X]Schizoaffective psychosis, manic type
Eu25012	[X]Schizophreniform psychosis, manic type
Eu25111	[X]Schizoaffective psychosis, depressive type
Eu25112	[X]Schizophreniform psychosis, depressive type
Eu25212	[X]Mixed schizophrenic and affective psychosis
Eu25z11	[X]Schizoaffective psychosis NOS
Eu26.00	[X]Nonorganic psychosis in remission
Eu2y.11	[X]Chronic hallucinatory psychosis
Eu2z.00	[X]Unspecified nonorganic psychosis
Eu2z.11	[X]Psychosis NOS
Eu31.12	[X]Manic-depressive psychosis
Eu32312	[X]Single episode of psychogenic depressive psychosis
Eu32314	[X]Single episode of reactive depressive psychosis
Eu33213	[X]Manic-depress psychosis,depressd,no psychotic symptoms
Eu33312	[X]Manic-depress psychosis,depressed type+psychotic symptoms
Eu33314	[X]Recurr severe episodes/psychogenic depressive psychosis
Eu33316	[X]Recurrent severe episodes/reactive depressive psychosis
Eu3z.11	[X]Affective psychosis NOS
Eu44.14	[X]Hysterical psychosis
Eu53111	[X]Puerperal psychosis NOS
Eu84013	[X]Infantile psychosis
Eu84111	[X]Atypical childhood psychosis
Eu84312	[X]Disintegrative psychosis
Eu84314	[X]Symbiotic psychosis
R20..00	[D]Senility, without mention of psychosis
R20z.00	[D]Senility, without psychosis NOS
ZV11111	[V]Personal history of manic-depressive psychosis
ZV11112	[V]Personal history of manic-depressive psychosis
212W.00	Schizophrenia resolved
E14z.11	Childhood schizophrenia NOS

Medical code	Description
Eu05200	[X]Organic delusional [schizophrenia-like] disorder
Eu2..00	[X]Schizophrenia, schizotypal and delusional disorders
Eu20.00	[X]Schizophrenia
Eu20000	[X]Paranoid schizophrenia
Eu20011	[X]Paraphrenic schizophrenia
Eu20100	[X]Hebephrenic schizophrenia
Eu20111	[X]Disorganised schizophrenia
Eu20200	[X]Catatonic schizophrenia
Eu20300	[X]Undifferentiated schizophrenia
Eu20311	[X]Atypical schizophrenia
Eu20500	[X]Residual schizophrenia
Eu20511	[X]Chronic undifferentiated schizophrenia
Eu20600	[X]Simple schizophrenia
Eu20y00	[X]Other schizophrenia
Eu20y11	[X]Cenesthopathic schizophrenia
Eu20z00	[X]Schizophrenia, unspecified
Eu21.12	[X]Borderline schizophrenia
Eu21.13	[X]Latent schizophrenia
Eu21.14	[X]Prepsychotic schizophrenia
Eu21.15	[X]Prodromal schizophrenia
Eu21.16	[X]Pseudoneurotic schizophrenia
Eu21.17	[X]Pseudopsychopathic schizophrenia
Eu23111	[X]Bouffee delirante with symptoms of schizophrenia
Eu23200	[X]Acute schizophrenia-like psychotic disorder
Eu25211	[X]Cyclic schizophrenia
ZV11000	[V]Personal history of schizophrenia
285..11	Psychotic condition, insight present
286..11	Poor insight into psychotic condition
38C1300	Assessment of psychotic and behavioural symptoms of dementia
38C1400	Assessment of cause of psychotic and behavioural symptoms
38Qh.00	Glasgow Antipsychotic Side-effect Scale
69DD.00	Antipsychotic medication physical health check

Medical code	Description
8BM0100	Antipsychotic medication review
8BPa.00	Antipsychotic drug therapy for dementia
8BPf.00	Oral antipsychotic therapy
E0...00	Organic psychotic conditions
E00..00	Senile and presenile organic psychotic conditions
E130.11	Psychotic reactive depression
E13z.11	Psychotic episode NOS
E2...00	Neurotic, personality and other nonpsychotic disorders
E2A..00	Nonpsychotic mental disorders following organic brain damage
E2Az.00	Nonpsychotic mental disorder post-organic brain damage NOS
Eu10500	[X]Mental & behav dis due to use alcohol: psychotic disorder
Eu11500	[X]Mental & behav dis due to use opioids: psychotic disorder
Eu12500	[X]Mental & behav dis due to cannabinoids: psychotic disordr
Eu13500	[X]Mental & behav dis due to seds/hypntcs: psychotic disordr
Eu14500	[X]Mental & behav dis due to use cocaine: psychotic disorder
Eu15500	[X]Mental/behav dis oth stims inc caffeine: psychotic dis
Eu16500	[X]Mental & behav dis due to hallucinogens: psychotic disord
Eu17500	[X]Mental & behav dis due to use tobacco: psychotic disorder
Eu18500	[X]Mental & behav dis due to vol solvents: psychotic disordr
Eu18700	[X]Mnt/bh dis vol solvents: resid & late-onset psychotic dis
Eu1A500	[X]Mental behav disord due crack cocaine: psychotic disorder
Eu23.00	[X]Acute and transient psychotic disorders
Eu23300	[X]Other acute predominantly delusional psychotic disorders
Eu23y00	[X]Other acute and transient psychotic disorders
Eu23z00	[X]Acute and transient psychotic disorder, unspecified

Medical code	Description
Eu24.13	[X]Induced psychotic disorder
Eu2y.00	[X]Other nonorganic psychotic disorders
Eu30100	[X]Mania without psychotic symptoms
Eu30200	[X]Mania with psychotic symptoms
Eu30211	[X]Mania with mood-congruent psychotic symptoms
Eu30212	[X]Mania with mood-incongruent psychotic symptoms
Eu31100	[X]Bipolar affect disorder cur epi manic wout psychotic symp
Eu31200	[X]Bipolar affect disorder cur epi manic with psychotic symp
Eu32200	[X]Severe depressive episode without psychotic symptoms
Eu32211	[X]Single episode agitated depressn w'out psychotic symptoms
Eu32212	[X]Single episode major depression w'out psychotic symptoms
Eu32213	[X]Single episode vital depression w'out psychotic symptoms
Eu32300	[X]Severe depressive episode with psychotic symptoms
Eu32311	[X]Single episode of major depression and psychotic symptoms
Eu32313	[X]Single episode of psychotic depression
Eu32700	[X]Major depression, severe without psychotic symptoms
Eu32800	[X]Major depression, severe with psychotic symptoms
Eu33211	[X]Endogenous depression without psychotic symptoms
Eu33212	[X]Major depression, recurrent without psychotic symptoms
Eu33214	[X]Vital depression, recurrent without psychotic symptoms
Eu33311	[X]Endogenous depression with psychotic symptoms
Eu33313	[X]Recurr severe episodes/major depression+psychotic symptom
Eu33315	[X]Recurrent severe episodes of psychotic depression
SL93.00	Other antipsychotics/neuroleptics/tranquilliser poisoning
SLX..00	Poisoning by oth & unspec antipsychotics & neuroleptics
SyuFL00	[X]Poisoning by oth & unspec antipsychotics & neuroleptics

Medical code	Description
E11..11	Bipolar psychoses
E11..13	Manic psychoses
E110.00	Manic disorder, single episode
E110.11	Hypomanic psychoses
E110000	Single manic episode, unspecified
E110100	Single manic episode, mild
E110200	Single manic episode, moderate
E110500	Single manic episode in partial or unspecified remission
E110600	Single manic episode in full remission
E110z00	Manic disorder, single episode NOS
E111.00	Recurrent manic episodes
E111000	Recurrent manic episodes, unspecified
E111100	Recurrent manic episodes, mild
E111200	Recurrent manic episodes, moderate
E111500	Recurrent manic episodes, partial or unspecified remission
E111600	Recurrent manic episodes, in full remission
E111z00	Recurrent manic episode NOS
E114.00	Bipolar affective disorder, currently manic
E114.11	Manic-depressive - now manic
E114000	Bipolar affective disorder, currently manic, unspecified
E114100	Bipolar affective disorder, currently manic, mild
E114200	Bipolar affective disorder, currently manic, moderate
E114500	Bipolar affect disord,currently manic, part/unspec remission
E114600	Bipolar affective disorder, currently manic, full remission
E114z00	Bipolar affective disorder, currently manic, NOS
E115.00	Bipolar affective disorder, currently depressed
E115.11	Manic-depressive - now depressed
E115000	Bipolar affective disorder, currently depressed, unspecified
E115100	Bipolar affective disorder, currently depressed, mild
E115200	Bipolar affective disorder, currently depressed, moderate

Medical code	Description
E115500	Bipolar affect disord, now depressed, part/unspec remission
E115600	Bipolar affective disorder, now depressed, in full remission
E115z00	Bipolar affective disorder, currently depressed, NOS
E116.00	Mixed bipolar affective disorder
E116000	Mixed bipolar affective disorder, unspecified
E116100	Mixed bipolar affective disorder, mild
E116200	Mixed bipolar affective disorder, moderate
E116500	Mixed bipolar affective disorder, partial/unspec remission
E116600	Mixed bipolar affective disorder, in full remission
E116z00	Mixed bipolar affective disorder, NOS
E117.00	Unspecified bipolar affective disorder
E117000	Unspecified bipolar affective disorder, unspecified
E117100	Unspecified bipolar affective disorder, mild
E117200	Unspecified bipolar affective disorder, moderate
E117500	Unspecified bipolar affect disord, partial/unspec remission
E117600	Unspecified bipolar affective disorder, in full remission
E117z00	Unspecified bipolar affective disorder, NOS
E11y.00	Other and unspecified manic-depressive psychoses
E11y000	Unspecified manic-depressive psychoses
E11y100	Atypical manic disorder
E11y300	Other mixed manic-depressive psychoses
E11yz00	Other and unspecified manic-depressive psychoses NOS
E112.11	Agitated depression
E112.12	Endogenous depression first episode
E112.13	Endogenous depression first episode
E112.14	Endogenous depression
E113.11	Endogenous depression - recurrent
E113700	Recurrent depression
E12..00	Paranoid states
E120.00	Simple paranoid state
E121.11	Sander's disease

Medical code	Description
E122.00	Paraphrenia
E123.00	Shared paranoid disorder
E123.11	Folie a deux
E12y.00	Other paranoid states
E12y000	Paranoia querulans
E12yz00	Other paranoid states NOS
E13..00	Other nonorganic psychoses
E13..11	Reactive psychoses
E132.00	Reactive confusion
E133.00	Acute paranoid reaction
E133.11	Bouffee delirante
E135.00	Agitated depression
E13y.00	Other reactive psychoses
E13y000	Psychogenic stupor
E13yz00	Other reactive psychoses NOS
E21..00	Personality disorders
E21..11	Neurotic personality disorder
E210.00	Paranoid personality disorder
E210.11	Fanatic personality
E211.00	Affective personality disorder
E211000	Unspecified affective personality disorder
E211100	Hypomanic personality disorder
E211200	Depressive personality disorder
E211300	Cyclothymic personality disorder
E211z00	Affective personality disorder NOS
E212.00	Schizoid personality disorder
E212000	Unspecified schizoid personality disorder
E212100	Introverted personality
E212200	Schizotypal personality
E212z00	Schizoid personality disorder NOS
E213.00	Explosive personality disorder
E213.11	Aggressive personality
E213.12	Quarrelsome personality

Medical code	Description
E214.00	Compulsive personality disorders
E214.11	Anancastic personality
E214000	Anankastic personality
E214100	Obsessional personality
E214z00	Compulsive personality disorder NOS
E215.00	Histrionic personality disorders
E215.11	Hysterical personality disorders
E215000	Unspecified histrionic personality disorder
E215100	Munchausen's syndrome
E215200	Emotionally unstable personality
E215300	Psychoinfantile personality
E215z00	Histrionic personality disorder NOS
E216.00	Inadequate personality disorder
E216.11	Asthenic personality
E216.12	Dependent personality
E216.13	Labile personality
E217.00	Antisocial or sociopathic personality disorder
E217.11	Amoral personality
E21y.00	Other personality disorders
E21y000	Narcissistic personality disorder
E21y100	Avoidant personality disorder
E21y200	Borderline personality disorder
E21y300	Passive-aggressive personality disorder
E21y400	Eccentric personality disorder
E21y500	Immature personality disorder
E21y600	Masochistic personality disorder
E21y700	Psychoneurotic personality disorder
E21y711	Neurotic personality
E21yz00	Other personality disorder NOS
E21yz11	Manipulative personality
E21z.00	Personality disorder NOS
E21z.11	Psychopathic personality
Eu20211	[X]Catatonic stupor

Medical code	Description
Eu20212	[X]Schizophrenic catalepsy
Eu20213	[X]Schizophrenic catatonia
Eu20214	[X]Schizophrenic flexibilatis cerea
Eu20400	[X]Post-schizophrenic depression
Eu20512	[X]Restzustand schizophrenic
Eu20y12	[X]Schizophreniform disord NOS
Eu20y13	[X]Schizophrenifrm psychos NOS
Eu21.00	[X]Schizotypal disorder
Eu21.11	[X]Latent schizophrenic reaction
Eu21.18	[X]Schizotypal personality disorder
Eu22.00	[X]Persistent delusional disorders
Eu22000	[X]Delusional disorder
Eu22012	[X]Paranoid state
Eu22013	[X]Paraphrenia - late
Eu22014	[X]Sensitiver Beziehungswahn
Eu22015	[X]Paranoia
Eu22100	[X]Delusional misidentification syndrome
Eu22111	[X]Capgras syndrome
Eu22200	[X]Cotard syndrome
Eu22300	[X]Paranoid state in remission
Eu22y00	[X]Other persistent delusional disorders
Eu22y11	[X]Delusional dysmorphophobia
Eu22y12	[X]Involutional paranoid state
Eu22y13	[X]Paranoia querulans
Eu22z00	[X]Persistent delusional disorder, unspecified
Eu23000	[X]Acute polymorphic psychot disord without symp of schizoph
Eu23011	[X]Bouffee delirante
Eu23100	[X]Acute polymorphic psychot disord with symp of schizophren
Eu23211	[X]Brief schizophreniform disorder
Eu23212	[X]Brief schizophrenifrm psych
Eu23213	[X]Oneirophrenia
Eu23214	[X]Schizophrenic reaction

Medical code	Description
Eu24.00	[X]Induced delusional disorder
Eu24.11	[X]Folie a deux
Eu24.12	[X]Induced paranoid disorder
Eu25.00	[X]Schizoaffective disorders
Eu25000	[X]Schizoaffective disorder, manic type
Eu25100	[X]Schizoaffective disorder, depressive type
Eu25200	[X]Schizoaffective disorder, mixed type
Eu25y00	[X]Other schizoaffective disorders
Eu25z00	[X]Schizoaffective disorder, unspecified
Eu30.00	[X]Manic episode
Eu30.11	[X]Bipolar disorder, single manic episode
Eu30000	[X]Hypomania
Eu30213	[X]Manic stupor
Eu30y00	[X]Other manic episodes
Eu30z00	[X]Manic episode, unspecified
Eu30z11	[X]Mania NOS
Eu31.00	[X]Bipolar affective disorder
Eu31.11	[X]Manic-depressive illness
Eu31.13	[X]Manic-depressive reaction
Eu31000	[X]Bipolar affective disorder, current episode hypomanic
Eu31300	[X]Bipolar affect disorder cur epi mild or moderate depressn
Eu31400	[X]Bipol aff disord, curr epis sev depress, no psychot symp
Eu31500	[X]Bipolar affect dis cur epi severe depres with psyc symp
Eu31600	[X]Bipolar affective disorder, current episode mixed
Eu31700	[X]Bipolar affective disorder, currently in remission
Eu31800	[X]Bipolar affective disorder type I
Eu31900	[X]Bipolar affective disorder type II
Eu31911	[X]Bipolar II disorder
Eu31y00	[X]Other bipolar affective disorders
Eu31y11	[X]Bipolar II disorder
Eu31y12	[X]Recurrent manic episodes
Eu31z00	[X]Bipolar affective disorder, unspecified

Medical code	Description
Eu32.00	[X]Depressive episode
Eu32.11	[X]Single episode of depressive reaction
Eu32.12	[X]Single episode of psychogenic depression
Eu32.13	[X]Single episode of reactive depression
Eu32000	[X]Mild depressive episode
Eu32100	[X]Moderate depressive episode
Eu32400	[X]Mild depression
Eu32500	[X]Major depression, mild
Eu32600	[X]Major depression, moderately severe
Eu32900	[X]Single major depr ep, severe with psych, psych in remiss
Eu32A00	[X]Recurr major depr ep, severe with psych, psych in remiss
Eu32B00	[X]Antenatal depression
Eu32y00	[X]Other depressive episodes
Eu32y11	[X]Atypical depression
Eu32y12	[X]Single episode of masked depression NOS
Eu32z00	[X]Depressive episode, unspecified
Eu32z11	[X]Depression NOS
Eu32z12	[X]Depressive disorder NOS
Eu32z13	[X]Prolonged single episode of reactive depression
Eu32z14	[X] Reactive depression NOS
Eu33.00	[X]Recurrent depressive disorder
Eu33.11	[X]Recurrent episodes of depressive reaction
Eu33.12	[X]Recurrent episodes of psychogenic depression
Eu33.13	[X]Recurrent episodes of reactive depression
Eu33.14	[X]Seasonal depressive disorder
Eu33.15	[X]SAD - Seasonal affective disorder
Eu33000	[X]Recurrent depressive disorder, current episode mild
Eu33100	[X]Recurrent depressive disorder, current episode moderate
Eu33200	[X]Recurr depress disorder cur epi severe without psyc sympt
Eu33300	[X]Recurrent depress disorder cur epi severe with psyc symp

Medical code	Description
Eu33400	[X]Recurrent depressive disorder, currently in remission
Eu33y00	[X]Other recurrent depressive disorders
Eu33z00	[X]Recurrent depressive disorder, unspecified
Eu33z11	[X]Monopolar depression NOS
Eu3z.00	[X]Unspecified mood affective disorder

Table S1-2: Drug Code List of antipsychotic drugs used for the data extraction

Drug	BNF code
Amisulpride	(0402010A0)
Aripiprazole	(0402010AD)
Benperidol	(0402010B0)
Cariprazine	(0402010AJ)
Chlorpromazine Hydrochloride	(0402010D0)
Chlorprothixene	(0402010F0)
Clozapine	(0402010C0)
Flupentixol Hydrochloride	(0402010H0)
Fluphenazine Hydrochloride	(0402010I0)
Haloperidol	(0402010J0)
Levomepromazine Hydrochloride	(0402010L0)
Levomepromazine Maleate	(0402010K0)
Loxapine Succinate	(0402010M0)
Lurasidone	(0402010AI)
Melperone Hydrochloride	(0402010AF)
Olanzapine	(40201060)
Paliperidone	(0402010AE)
Pericyazine	(0402010P0)
Perphenazine	(0402010Q0)
Pimozide	(0402010R0)
Promazine Hydrochloride	(0402010S0)
Quetiapine	(0402010AB)
Risperidone	(40201030)
Sulpiride	(0402010U0)

Thioridazine	(0402010W0)
Trifluoperazine	(0402010X0)
Ziprasidone Hydrochloride	(0402010AG)
Zotepine	(0402010AC)
Zuclopenthixol Acetate	(40201010)
Zuclopenthixol Hydrochloride	(0402010T0)

Supplemental material S2

Cardiometabolic monitoring trends for body composition (body weight, BMI); blood glucose; blood lipids by the associated cardiometabolic risks of antipsychotic.

Table S2-1: Trends in body compositions (body weight, BMI) monitoring performance by risks of weight gain associated with antipsychotic use.

BMI monitoring level	Weight gains associated risks with APs use					p-value†
	Limited/No data	Neutral/ low	Moderate	High	Total	
Never	13	6	35	12	66	0.2
Less frequent	15	29	77	33	154	
Standard	14	11	62	19	106	
More frequent	19	34	82	36	171	
Total	61	80	256	100	497	

†Results of chi-square ($\chi 2$) & Fisher exact tests; *** p<.01, ** p<.05, * p<.1; NR: No records

Table S2-2: Trends in HbA1c monitoring performance by risks of hyperglycemia/diabetes associated with APs use

HbA1c monitoring level	Hyperglycaemia/diabetes risks associated with APs					p-value†
	Limited/No data	Minimal	Low	Moderate	High	
Never	10	88	64	247	83	0.3
Less frequent	NR	2	NR	1	2	
Total	10	90	64	248	85	

†Results of chi-square ($\chi 2$) & Fisher exact tests; *** p<.01, ** p<.05, * p<.1; NR: No records

Table S2-3: Trends in lipid panel monitoring performance by risks dyslipidemia associated with APs use.

HDL monitoring level	Risks dyslipidaemia associated with APs use			
	Neutral/ Low	Moderate/High	Total	p-value†
Never	38	112	150	<0.00***
Less frequent	69	125	194	
Standard	22	51	73	
More frequent	43	36	80	
Total	172	324	497	
Non-HDL monitoring level	Neutral/ Low	Moderate/High	Total	p-value†
Never	46	125	171	<0.00***
Less frequent	67	125	192	
Standard	23	47	70	
More frequent	36	27	64	
Total	172	324	497	

†Results of chi-square ($\chi2$) & Fisher exact tests; *** p<.01, ** p<.05, * p<.1; NR: No records
